# Clozapine, chlorpromazine and risperidone dose-dependently reduce emotional hyperthermia, a biological marker of salience

**DOI:** 10.1007/s00213-017-4710-x

**Published:** 2017-08-15

**Authors:** William W. Blessing, Esther M. Blessing, Mazher Mohammed, Youichirou Ootsuka

**Affiliations:** 1 Center for Neuroscience, Department of Human Physiology, Flinders University, Adelaide, SA Australia; 2 Department of Psychiatry, New York University School of Medicine, New York, NY USA

**Keywords:** Antipsychotic drugs, Body temperature, Brown adipose tissue thermogenesis, Cutaneous blood flow, Stress-induced hyperthermia

## Abstract

**Rationale:**

We recently introduced a new rat model of emotional hyperthermia in which a salient stimulus activates brown adipose tissue (BAT) thermogenesis and tail artery constriction. Antipsychotic drugs, both classical and second generation, act to reduce excessive assignment of salience to objects and events in the external environment. The close association between salient occurrences and increases in body temperature suggests that antipsychotic drugs may also reduce emotional hyperthermia.

**Objectives:**

We determined whether chlorpromazine, clozapine, and risperidone dose dependently reduce emotionally elicited increases in BAT thermogenesis, cutaneous vasoconstriction, and body temperature in rats.

**Methods:**

Rats, chronically instrumented for measurement of BAT and body temperature and tail artery blood flow, singly housed, were confronted with an intruder rat (confined within a small wire-mesh cage) after systemic pre-treatment of the resident rat with vehicle or antipsychotic agent. BAT and body temperatures, tail blood flow, and behavioral activity were continuously measured.

**Results:**

Clozapine (30 μg–2 mg/kg), chlorpromazine (0.1–5 mg/kg), and risperidone (6.25 μg–1 mg/kg) robustly and dose-relatedly reduced intruder-elicited BAT thermogenesis and tail artery vasoconstriction, with consequent dose-related reduction in emotional hyperthermia.

**Conclusions:**

Chlorpromazine, a first-generation antipsychotic, as well as clozapine and risperidone, second-generation agents, dose-dependently reduce emotional hyperthermia. Dopamine D_2_ receptor antagonist properties of chlorpromazine do not contribute to thermoregulatory effects. Interactions with monoamine receptors are important, and these monoamine receptor interactions may also contribute to the therapeutic effects of all three antipsychotics. Thermoregulatory actions of putative antipsychotic agents may constitute a biological marker of their therapeutic properties.

## Introduction

Inappropriate attribution of salience to insignificant stimuli is a core feature of the psychotic process in schizophrenia, contributing to impaired attention and fragmented consciousness, as well as to positive symptoms, including delusions (Barkus et al. 2014; Jensen and Kapur 2009; Kapur 2003; McGhie and Chapman 1961; Roiser et al. 2013). Effects of antipsychotic agents on salience-related biological processes have been assessed principally for pre-pulse inhibition of the startle response to a sudden unexpected sound (PPI) (Braff and Geyer 1990). An additional model in which antipsychotic agents, both classical and second generation, have consistent robust dose-dependent effects on a separate salience-related physiological process would be valuable, especially if, like PPI, the model is applicable to both laboratory animals and humans.

Detection of a salient, emotionally significant, event increases body temperature by increasing heat production and by decreasing heat dissipation. Brown adipose tissue (BAT) is activated (Mohammed et al. 2014), and the thermoregulatory cutaneous vascular bed constricts (we go pale with fright) (Blessing et al. 2016; Mittelmann and Wolff 1943). Because BAT thermogenesis and cutaneous vasoconstriction are directly controlled by sympathetic outflow from the central nervous system (CNS), emotional hyperthermia is a primary physiological event, not secondary to behavioral activity triggered by the salient event. Threatening or frankly stressful events are more potent stimuli for BAT thermogenesis, but any novel or unexpected event increases cutaneous sympathetic discharge, as documented in humans (Delius et al. 1972).

The term “emotional hyperthermia” was first popularized and the phenomenon experimentally documented in humans by Renbourn (1960) who discussed the possible contributions to the hyperthermia of reduced heat transfer to the environment versus increased basal metabolic rate. The demonstration of active BAT metabolism in adult humans (Cypess et al. 2009; Nedergaard et al. 2007; Saito et al. 2009; van Marken Lichtenbelt et al. 2009; Virtanen et al. 2009) raises the possibility that heat production in BAT could contribute to emotional hyperthermia in humans. Indeed, mild psychological stress has now been demonstrated to activate BAT thermogenesis in adult humans (Robinson et al. 2016).

Chlorpromazine, the first effective antipsychotic drug, was introduced into psychiatry after initial use to reduce body temperature during major surgical procedures, a process referred to as “artificial hibernation” (Lopez-Munoz et al. 2005). The underlying physiological mechanisms, extensively studied last century, include both increased heat loss from the body via the cutaneous circulation and decreased basal metabolic rate [see references from Blessing et al. (2016))]. Clozapine, the gold standard second-generation antipsychotic agent and risperidone, a commonly used second-generation drug, also reduce body temperature in humans (Heh et al. 1988; van Marum et al. 2007) and clozapine powerfully dilates the cutaneous vascular bed in humans with schizophrenia and schizoaffective disorders (Blessing et al. 2011). In laboratory animals, clozapine and risperidone reduce body temperature, and clozapine dilates the thermoregulatory cutaneous circulation and decreases BAT thermogenesis, as does the widely used second-generation agent olanzapine [see references from Blessing et al. (2016))].

Remarkably, therefore, both first- and second-generation antipsychotic agents reduce body temperature in both animals and humans by modulating both arms of the thermoregulatory response: heat transfer and heat production. Effects of antipsychotic agents on the mechanisms underlying emotional hyperthermia have not been reported in experimental animals or in humans. Robust thermoregulatory effects might provide a new model for the evaluation of putative antipsychotic agents, particularly because much is known concerning CNS pathways and neurotransmitters controlling heat production and heat transfer (Blessing et al. 2016; Morrison and Madden 2014).

In our recently described experimental model of emotional hyperthermia (Mohammed et al. 2014), a conscious unrestrained rat, maintained in an isolated temperature-controlled ad libitum food environment (resident rat), is suddenly and unexpectedly confronted with an intruder rat (confined to a small wire-mesh cage), an intensely salient event that strongly activates thermoregulatory cutaneous vasoconstriction and BAT thermogenesis in the resident rat. Since there is no actual physical contact between intruder and resident animals, the stimulus is psychological rather than physical.

In our present intruder rat study, BAT and body temperatures, cutaneous blood flow, and behavioral activity were measured simultaneously in the conscious freely moving resident rat. We determined whether pre-treatment with clozapine, chlorpromazine, or risperidone dose-dependently reduce the degree of emotional hyperthermia elicited by the intrusion, and whether reduced BAT thermogenesis and reduced cutaneous vasoconstriction contributes to the antipsychotic-mediated effect. In addition, we determined the effect of raclopride (a selective dopamine D_2_ receptor antagonist) on resting cutaneous blood flow.

## Materials and methods

### Intruder rat model

Our intruder rat experimental model has been fully described (Mohammed et al. 2014). For implantation of measuring devices, rats were anesthetized with 2% isoflurane (Veterinary Companies of Australia Pty, Ltd., NSW, Australia) in oxygen. Thermistor probes were positioned in interscapular BAT near the vein of Sulzer (BAT temperature) and in the anterior mediastinum ventral to the trachea (body temperature). A Doppler ultrasonic probe (Iowa Doppler Products, Iowa City, USA) was chronically implanted around the base of the tail artery, the principal thermoregulatory cutaneous vascular bed in rats (Rand et al. 1965). Insulated wires from the temperature probes and the tail artery Doppler probes were passed subcutaneously and attached to a head socket. After recovery from anesthesia, the animal was returned to the animal house and individually caged for at least 1 week before experiments were carried out.

The day before the first experiment, the rat (resident rat) was transferred to a plastic-walled open-roofed cage (40-cm long, 35-cm wide, 45-cm high) with food and water ad libitum. A head socket was connected to recording devices via a flexible cable and counter-balanced swivel (SL12C, PlasticsOne, Roanoke, VA, USA) positioned above the cage. The animal was fitted with a chronic subcutaneous or intraperitoneal catheter, with the connecting tubing accessible at the swivel for administration of drugs. The cage was isolated within a modified commercial freezer, maintained at 24–26 °C, with lights off at 0700 h, and lights on at 1900 h. This minimized exposure to environmental disturbances.

Temperature signals were passed to a bridge amplifier (Biomedical Engineering, Flinders University) and then digitized (1 Hz sampling rate) with PowerLab (ADInstruments, Castle Hill, NSW, Australia). The pulsatile tail artery Doppler flow signal was passed to an analyzer (Model 200-202, Triton Technology, San Diego, CA, USA) and then digitized (40 Hz sampling rate) with PowerLab using the Chart software. Behavioral activity was measured by quantifying (10 Hz sampling rate) movement-related changes in infrared signal emitted from the resident rat. Experimental parameters were continuously recorded.

We have previously documented that BAT and body temperatures increase in an episodic ultradian manner approximately every 1–2 h (Blessing and Ootsuka 2016; Ootsuka et al. 2009), emphasizing the importance of selecting an appropriate baseline in experimental studies. At a time when BAT temperature was near baseline, either vehicle or drug (see the following sections) was administered. After 30 min, a second male Sprague-Dawley rat (intruder rat) confined to a small wire-mesh cage was suddenly introduced into the cage of the resident rat. Experimental parameters were recorded for an additional 30 min and then the intruder rat was removed.

### Drugs

Drugs were administered subcutaneously or intraperitoneally via the chronically implanted catheter (see previous section). The catheter was first flushed with 0.5 ml Ringer, and then, the drug was administered in 0.5 ml of vehicle, and then the catheter was again flushed with 0.5 ml of Ringer. Clozapine (Tocris Bioscience) 30 μg–2 mg/kg and risperidone 6.25 μg–1 mg/kg (Abcam Biochemicals) were dissolved in acidified Ringer. Chlorpromazine, (Sigma-Aldrich) 0.1–5 mg/kg, was dissolved in water. The dose of raclopride (Sigma-Aldrich 0.5 mg/kg, dissolved in Ringer) was selected from the literature (Swerdlow et al. 1991).

Each resident rat was used for a maximum of four experimental conditions, with at least 3 days between each administration of vehicle or a given drug dose. We have previously shown that the response to the intruder rat does not habituate under these conditions (Mohammed et al. 2014). Experiments for each drug were conducted separately, using a rotating dose design to control for serial effects.

### Data analysis

The PowerLab Chart files were imported into Igor Pro (WaveMetrics, Portland, OR, USA) for organization and graphical presentation of results. Statistical analysis was performed using Statview (SAS institute, Carey, NC, USA). Group results (mean ± SEM) were calculated for vehicle and for each drug dose.

To evaluate the effects of drugs on BAT and body temperature, we calculated the difference between the mean of the 5 min period before drug administration and the mean of the 5 min period from 23 to 28 min after drug administration (delta BAT and body temperatures). For tail artery blood flow, we calculated the mean of the 5-min period from 23 to 28 min after drug administration.

To evaluate the effect of the intruder for vehicle and each drug dose in each rat, we calculated the change in BAT and body temperatures for the 5 min period before introduction of the intruder compared with the values 18–28 min after introduction of the intruder (delta BAT and body temperatures). The longer time period was selected because of interanimal variation in the timing of intruder-elicited changes in temperature. For tail artery blood flow and behavioral activity, we calculated the mean value for the initial 5 min after introduction of the intruder rat. For behavioral activity, we calculated the percentage of the initial 5 min post-introduction of the intruder time during which the animal was moving.

In the intruder situation, for vehicle-treated rats, we used a paired “t” test to compare the slope of the BAT versus body temperature traces during the first 5 min after introduction of the intruder, as well as the change in BAT versus body temperature measured 18–28 min after introduction of the intruder. If BAT temperature is a valid measure of BAT thermogenesis, as we established in our previous study (Mohammed et al. 2014), then the intruder-elicited BAT temperature parameters should be greater than the corresponding body temperature parameters.

For each antipsychotic agent (separately) and for each measured parameter, we pooled the intruder-elicited results for all the drug doses and calculated the Pearson correlation coefficient between the BAT temperature slope and the tail artery blood flow 0–5 min post-introduction of the intruder.

Dose-response relations for each drug treatment and for each variable were assessed using linear regression between log dose and the measured parameter. If a given regression was not significant, we then used factorial ANOVA to determine if the parameter after a particular dose of an antipsychotic agent was different from vehicle. In this case, we used Fisher’s least significant difference (LSD) for post hoc analysis. For each variable assessed in the intruder situation, we used factorial ANOVA to determine whether the values observed after the lowest dose of each antipsychotic agent were significantly different from vehicle. The significance level for all statistical evaluations was set at *P* < 0.05.

## Results

### Effect of antipsychotic agents on baseline thermoregulatory variables

**Fig. 1:**
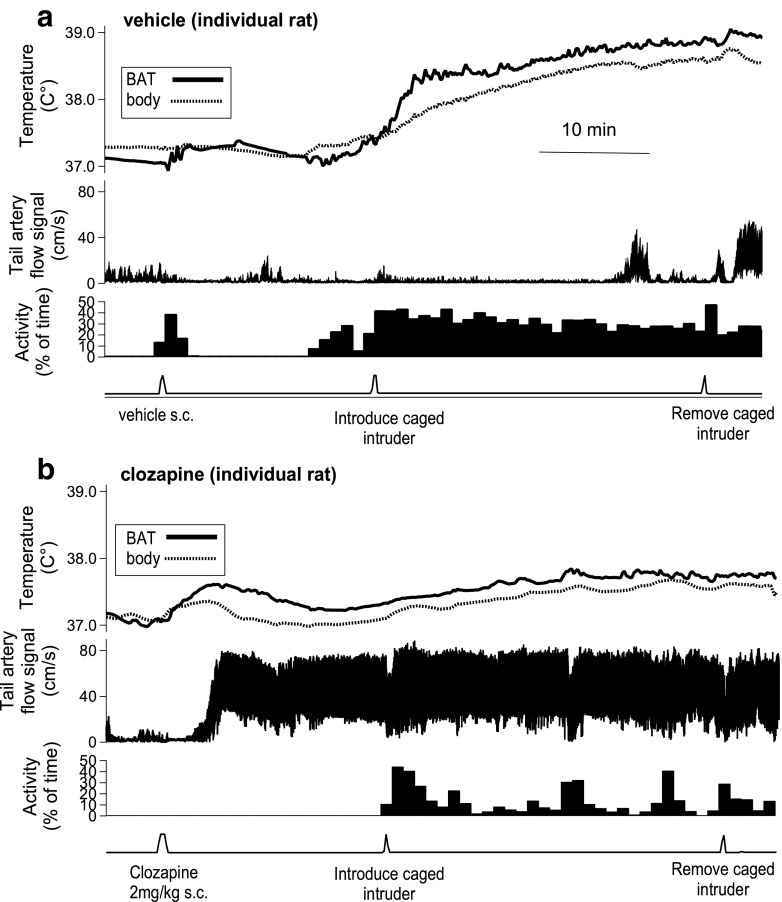
Original experimental records from individual rats before and after administration of vehicle (**a**) or clozapine (**b**) showing BAT and body temperatures (upper panels), tail artery Doppler flow signal (middle panels), and behavioral activity (lower panels). The caged intruder rat was introduced into and removed from the cage of the resident rat at the indicated times

**Fig. 2:**
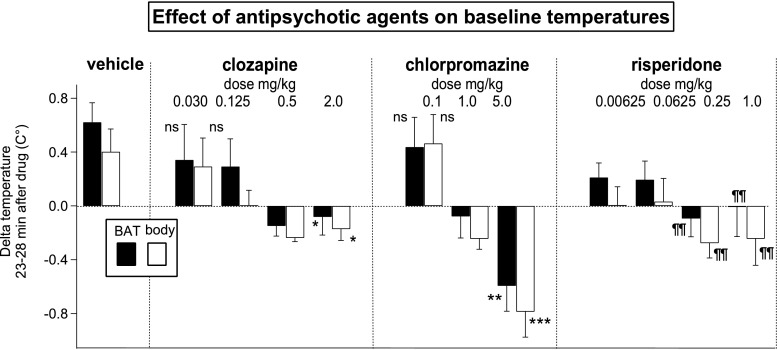
Bar graphs showing group results (mean ± SEM) of change in BAT (black bars) and body (white bars) temperatures measured in the resident rat 23–28 min after administration of vehicle or antipsychotic drug. *ns* Change in temperature for lowest dose of each drug not significantly different from vehicle, *P* > 0.05. Asterisks indicate significant linear regression between log dose of drug and drug-induced changes in BAT or body temperature signals, *P < 0.05, **P < 0.01, ***P < 0.001. Risperidone log dose regression not significant; *P* > 0.05 for both BAT and body delta temp; ¶¶ significantly different from change in BAT or body temp value after vehicle, *P* < 0.01

Recordings from the resident rat after administration of drug and subsequent introduction of a caged intruder rat are shown in Fig. 1a (vehicle), b (clozapine 2 mg/kg). Group results for changes in BAT and body temperature measured 23–28 min after vehicle and increasing doses of each antipsychotic agent are shown in Fig. 2. Baseline BAT and body temperatures increased after vehicle or the lowest dose of each antipsychotic, changes related to the process of disturbing the animal for administration of drugs (Mohammed et al. 2014). Higher doses of all antipsychotic agents reversed these increases (in a dose-dependent manner for clozapine and chlorpromazine), so that for the highest dose of each antipsychotic agent, the 23–28 min post-drug BAT and body temperatures were less than the pre-administration values. For clozapine, log-dose regression for delta BAT temperature, *F*(1, 20) = 4.308, *P* = 0.051, *R* ^2^ = 0.18, and log-dose regression for delta body temperature, *F*(1, 17) = 7.811, *P* < 0.05, *R* ^2^ = 0.30. For chlorpromazine, log-dose regression for delta BAT temperature, *F*(1, 15) = 13.142, *P* < 0.01, *R* ^2^ = 0.47, and log-dose regression for delta body temperature *F*(1, 17) = 27.230, *P* < 0.0001, *R* ^2^ = 0.62. For risperidone, the log-dose regressions for both delta BAT and delta body temperatures were not significant (*P* > 0.05). Each dose was then compared to vehicle using factorial ANOVA and LSD post hoc analysis. For the two higher doses of risperidone (0.25 and 1.0 mg/kg), delta BAT temperature and delta body temperatures were lower than the corresponding values for vehicle (*P* < 0.01).

Clozapine increased resting tail artery blood flow in the present study (Fig. 1b), as we have previously reported for both clozapine and chlorpromazine (Blessing and Ootsuka 2007). In the present study, risperidone (0.0625, 0.25, and 1.0 mg/kg) also increased resting tail artery blood flow in a dose-dependent manner (significant log-dose linear regression, *F*(1, 14) = 79.795, *P* < 0.01, *R* ^2^ = 0.41). In contrast, the selective dopamine D_2_ antagonist raclopride (0.5 mg/kg) had no effect on resting tail artery blood flow (paired *t* = 0.395, *n* = 6, *P* > 0.05).

### Effect of antipsychotic agents on intruder-elicited thermoregulatory variables

#### Comparison of intruder-elicited increases in BAT versus body temperature after pre-treatment with vehicle

**Fig. 3:**
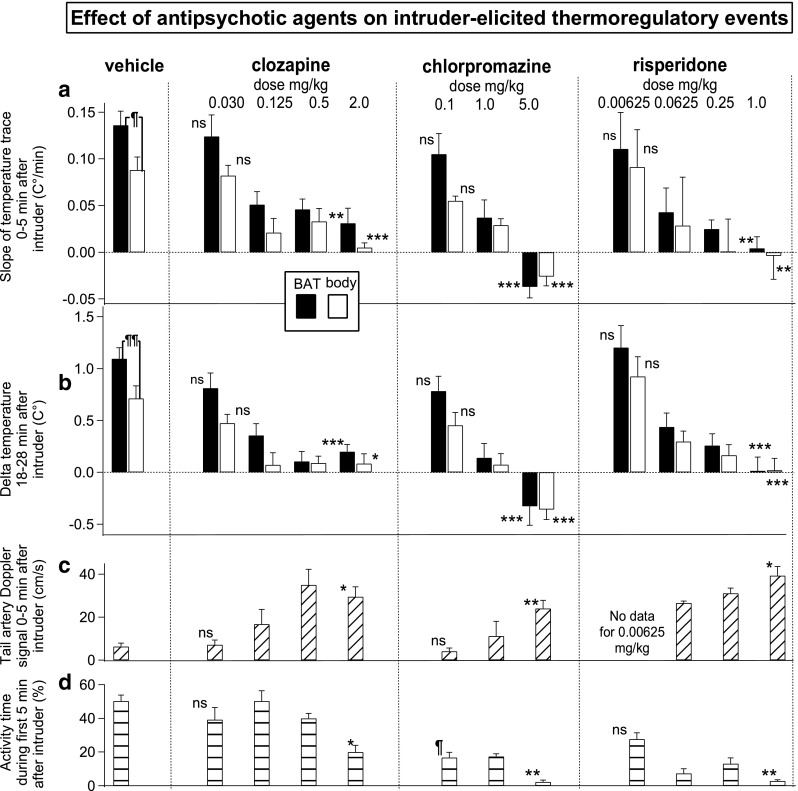
Bar graphs showing group results (mean ± SEM) of the effects of vehicle or antipsychotic drugs on intruder-elicited thermoregulatory events. **a** ¶ After vehicle pre-treatment, the intruder-elicited slope of the BAT (black bar) temperature signal is significantly greater than corresponding slope of the body (white bar) temperature signal (*P* < 0.05). *ns* lowest dose intruder-elicited 0–5 min slope of the temperature signals for each drug not significantly different from slope after vehicle, *P* > 0.05. Asterisks indicate significant linear regression between the log dose of drug and the slopes of the BAT or body temperature signals recorded from the resident rat during the first 5 min after introduction of the intruder rat, **P < 0.01, ***P < 0.001. **b** ¶¶ After vehicle pre-treatment, the intruder-elicited increase in BAT temperature (black bar) is significantly greater than corresponding increase in body temperature (white bar) (*P* < 0.01). *ns* lowest dose intruder-elicited increase in the temperature signal for each drug not significantly different from vehicle *P* > 0.05. Asterisks indicate significant linear regression between log dose of drug and changes in BAT or body temperature signals recorded from the resident rat 18–28 min after introduction of the intruder rat, *P < 0.05, ***P < 0.001. **c** *ns* lowest dose of each drug not significantly different from vehicle tail flow during the first 5 min after introduction of the intruder, *P* > 0.05. Asterisks indicate significant linear regression between log dose of drug and changes tail artery Doppler flow signal recorded from the resident rat 0–5 min after introduction of the intruder rat, *P < 0.05, **P < 0.01. **d** Behavioral activity during 0–5 min after introduction of the intruder rat (percent total time). *ns* lowest dose not significantly different from vehicle activity, *P* > 0.05. ¶ intruder-elicited activity after lowest dose of chlorpromazine significantly less than activity after vehicle. Asterisks indicate significant linear regression between log dose of drug and behavioral activity recorded from the resident rat 0–5 min after introduction of the intruder rat, *P < 0.05, **P < 0.01

Records of the effect of introducing the intruder rat after administration of vehicle are shown in Fig. 1a (vehicle), and group results are shown in Fig. 3. Both BAT and body temperatures increased rapidly in the resident rat after introduction of the intruder following pre-treatment with vehicle (Fig. 1a). In these animals, the 0–5 min post-introduction of the intruder slope of the BAT temperature records and the 18–28 min increase in BAT temperature were both greater than the corresponding values for body temperature (see Fig. 3a, b, first panels); for the slope of the BAT and body temperature records, paired *t* test = 2.960, *n* = 8, *P* < 0.05; for the delta BAT and body temperatures, paired *t* test = 3.931, *n* = 8, *P* < 0.01.

The higher values for BAT temperature in comparison with body temperature confirm that BAT thermogenesis contributes to the intruder-elicited increases in body temperature, in agreement with our previous study (Mohammed et al. 2014).

#### Comparison of intruder-elicited increases in BAT and body temperature after pre-treatment with vehicle or antipsychotic agents

For each drug examined, the post-introduction of the intruder slope of the BAT and body temperature records and the 18–28 min increases in BAT and body temperatures after the lowest dose of each drug were not significantly different from the corresponding vehicle values (Fig. 3a, b and figure legends).

In the example from an individual rat shown in Fig. 1b, the intruder-related increases in BAT and body temperature are substantially blunted by pre-treatment with clozapine. Group results (Fig. 3a, b) show that clozapine, chlorpromazine, and risperidone all dose-relatedly decreased the 0–5 min post-introduction of the intruder slopes of the BAT and body temperature records. For the BAT temperature slope: clozapine *F*(1, 21) = 13.218, *P* < 0.01, *R* ^2^ = 0.36; chlorpromazine *F*(1, 15) = 28.613, *P* < 0.0001, *R* ^2^ = 0.66; risperidone *F*(1, 21) = 14.074, *P* < 0.001, *R* ^2^ = 0.40. For the body temperature slope: clozapine *F*(1, 17) = 15.339, *P* < 0.01, *R* ^2^ = 0.15; chlorpromazine *F*(1, 17) = 52.508, *P* < 0.0001, *R* ^2^ = 0.76; risperidone *F*(1, 20) = 11.532, *P* < 0.01, *R* ^2^ = 0.37.

Similarly, all three antipsychotic agents dose-relatedly decreased the delta BAT and body temperatures. For delta BAT temperature: clozapine *F*(1, 21) = 14.890, *P* < 0.001, *R* ^2^ = 0.42; chlorpromazine *F*(1, 15) = 27.790, *P* < 0.0001, *R* ^2^ = 0.65; risperidone *F*(1, 21) = 31.771, *P* < 0.0001, *R* ^2^ = 0.602. For delta body temperature: clozapine *F*(1, 17) = 6.206, *P* < 0.05, *R* ^2^ = 0.27; chlorpromazine *F*(1, 17) = 34.818, *P* < 0.0001, *R* ^2^ = 0.67; risperidone *F*(1, 21) = 25.779, *P* < 0.0001, *R* ^2^ = 0.56.

#### Comparison of intruder-elicited deceases in amplitude of tail artery blood flow after pre-treatment with vehicle or antipsychotic agents

In the example from an individual rat in Fig. 1b, clozapine (2 mg/kg) completely prevents any intruder-elicited fall in tail artery blood flow. During the first 5 min after introduction of the intruder, tail artery blood flow values for the lowest doses of clozapine and chlorpromazine were not significantly different from vehicle (Fig. 3c). Clozapine, chlorpromazine, and risperidone all dose-relatedly prevented the intruder-elicited fall in tail artery blood flow (Fig. 3c): clozapine *F*(1, 17) = 7.146, *P* < 0.05, *R* ^2^ = 0.30; chlorpromazine *F*(1, 12) = 12.225, *P* < 0.01, *R* ^2^ = 0.51; risperidone *F*(1, 13) = 6.564, *P* < 0.05, *R* ^2^ = 0.34.

#### Comparison of intruder-elicited increases in behavioral activity after pre-treatment with vehicle or antipsychotic agents

When the intruder rat was introduced, resident rats pre-treated with vehicle became vigorously active, circling around and climbing on top of the intruder’s cage, as we previously reported (Mohammed et al. 2014). The activity values for the lowest doses of clozapine and risperidone were not significantly different from vehicle (Fig. 3d). Clozapine, chlorpromazine, and risperidone all dose-relatedly reduced the intruder-induced increase in activity (Fig. 3d); clozapine, *F*(1, 21) = 6.124, *P* < 0.05, *R* ^2^ = 0.23; chlorpromazine *F*(1, 16) = 9.040, *P* < 0.01, *R* ^2^ = 0.32; risperidone *F*(1, 22) = 16.339, *P* < 0.001, *R* ^2^ = 0.43. Especially for clozapine, this effect was modest, so that after the highest dose of clozapine (2 mg/kg), the resident animal was still actively moving around 20% of the total time.

#### Correlations between BAT temperature slope and tail artery blood flow during the first 5 min after introduction of the intruder

For each drug, we pooled the 0–5 min after introduction of the intruder for the slope of the BAT temperature record and the tail artery blood flow for all the doses of that drug. The Pearson correlation coefficients between the slope of the BAT temperature records and the tail artery blood flow during the first 5 min after introduction of the intruder were − 0.54 for clozapine (*P* < 0.05), − 0.67 for chlorpromazine (*P* < 0.05), and − 0.64 for risperidone (*P* < 0.05). Thus, the degree to which each antipsychotic agent reduced intruder-elicited BAT thermogenesis was significantly correlated with the degree to which it prevented the intruder-elicited constriction of the tail artery.

## Discussion

Chlorpromazine, a first-generation antipsychotic drug, and the second-generation drugs clozapine and risperidone all reduced emotional hyperthermia by inhibiting BAT thermogenesis and thermoregulatory cutaneous vasoconstriction. For each antipsychotic agent, the robustly dose-related variations in the intruder-elicited changes in BAT thermogenesis and tail artery vasoconstriction were highly correlated, attesting to the reliability and validity of our measurements. The substantial effects on thermoregulatory processes occurred without major impairment of the normal behavioral response to the emotional disturbance. Thus, the antipsychotic-mediated reduction of body temperature is not secondary to general sedative effects of the drugs.

Relatively low doses were required for major biological effects. Magnitudes of the mid-range effective dose for each of the three antipsychotic agents (approximately 0.1 mg/kg for risperidone, 0.2 mg/kg for clozapine, and 1 mg/kg for chlorpromazine) are of the same order as the relative magnitudes of their effective clinical therapeutic doses (Woods 2003). Clozapine abolished intruder-elicited increases in BAT thermogenesis and tail artery constriction at a dose of 0.5 mg/kg. This is less than the clozapine dose (1.5 mg/kg) required for similarly substantial effects on cardiovascular parameters induced by open field stress (van den Buuse 2003) and substantially less than doses of clozapine (4–20 mg/kg) used in studies of the acoustic startle response itself (Swerdlow et al. 1991) or in pre-pulse inhibition of this response (Geyer et al. 2001). Chlorpromazine had a substantial inhibitory effect on intruder-elicited BAT thermogenesis at a dose of 1.0 mg/kg, again much less than the doses (6–20 mg/kg) commonly used in experimental animal studies, including effects on PPI (Kollias and Bullard 1964; Swerdlow et al. 1998a). Reviews of the available evidence show that chlorpromazine, clozapine, and risperidone have inconsistent effects on PPI itself, and reversal by these antipsychotics of drug-induced deficits in PPI is also quite variable (Auclair et al. 2007; Geyer et al. 2001; Porsolt et al. 2010; Swerdlow et al. 1998b).

### Neurotransmitter receptors regulating thermoregulatory sympathetic outflows and cognitive/emotional function

Chlorpromazine, a first-generation agent, has inhibitory thermoregulatory actions that are remarkably similar to those of clozapine and risperidone, second-generation agents. However, in contrast to chlorpromazine, haloperidol, another important first-generation agent whose principal action is dopamine D_2_ receptor blockade, has little or no acute effect on resting body temperature in the rat (Chipkin 1988; Blessing 2004) and little or no effect on resting tail artery flow or on emotionally elicited tail artery vasoconstriction in the rat (Blessing 2005). The hypothermic action of apomorphine (D_2_ agonist) is dose-dependently prevented by pretreatment with haloperidol (Chipkin 1988). Low-dose spiperone (D_2_ antagonist) blocks the apomorphine-mediated increase in resting tail blood flow, the apomorphine-mediated inhibition of emotionally induced tail artery vasoconstriction, and the quinpirole-mediated (D_2_ agonist) inhibition of BAT thermogenesis in rats (Blessing and Ootsuka 2007; Ootsuka et al. 2007). In our present study, raclopride, another potent and selective D_2_ antagonist, had no effect on resting tail artery blood flow. Thus, stimulation, not blockade, of CNS dopamine D_2_ receptors lowers body temperature. Low-dose spiperone also reduces clozapine-mediated inhibition of tail artery vasoconstriction (Blessing and Ootsuka 2007) suggesting that D_2_ stimulation rather than D_2_ blockade contributes to the clozapine’s inhibitory thermoregulatory actions.

Since the dopamine D_2_ receptor blocking action of chlorpromazine is well established, the inhibitory thermoregulatory actions of this agent must be mediated by interactions with receptors other than the dopamine receptor. In experimental animals, 5-HT_1A_ agonists and 5-HT_2A_ antagonists substantially reduce the BAT thermogenesis and cutaneous vasoconstriction that contributes to emotional hyperthermia (Blessing et al. 2016; Morrison and Madden 2014). Clonidine, an alpha-2 adrenergic agonist, substantially reduces BAT thermogenesis (Madden et al. 2013) as well as emotionally induced constrictions of the rat tail artery (Mohammed et al. 2016). Activity of histamine-synthesizing neurons, via H_1_ receptors, also increases body temperature in association with activation of the ascending arousal system (Lkhagvasuren and Oka 2017; Valdes et al. 2010). It is likely that combinations of these receptor interactions contribute to the marked inhibitory thermoregulatory properties of clozapine and risperidone. Interactions with other monoamine receptors, together with non-monoamine receptors activated by acetylcholine, glutamate, cannabinoids, and neuropeptides may also mediate therapeutic effects of antipsychotic agents. Interactions with receptors associated with these neurotransmitters may also have marked thermoregulatory effects, as demonstrated, for example, for cannabinoid receptors (Lima et al. 2014; Smirnov and Kiyatkin 2008).

Given the close link between the activation of brain thermoregulatory control pathways and the emotional arousal associated with excessive salience, it is important to note that interactions with the same monoamine neurotransmitter receptors contribute to the therapeutic effects of second-generation antipsychotic agents (Brosda et al. 2014; Fitzgerald 2014; Kapur et al. 2005; Langer 2015; Meltzer 2013; Miyamoto et al. 2012; Newman-Tancredi et al. 1998). It may be that these monoamine interactions contribute to the antipsychotic actions of chlorpromazine as well as clozapine. There is evidence that this may be the case for 5-HT_2A_ receptor interactions (Trichard et al. 1998).

### The lateral habenula coordinates activity of monoamine neurons regulating both the behavioral and the autonomic components of the response to salient events

Output from the lateral habenula nucleus in the dorsal epithalamus integrates the monoamine-modulated response to unexpected, novel, often negative, salient events via direct projections to serotonin, noradrenaline, and histamine-synthesizing neurons in the upper brain stem, neurons with major ascending projections to forebrain centers, including the insula, the amygdala, the hippocampus, and the medial prefrontal cortex (Stephenson-Jones et al. 2012). These projections could be relevant to the involvement of 5-HT_1A_, 5-HT_2A_, alpha-2 adrenergic, and histamine H_1_ receptors in both thermoregulatory and cognitive/emotional functions. The insular cortex is important both for the affective component of temperature sensation and for attribution of salience (Craig et al. 2000; Palaniyappan et al. 2013). Dysfunction of the amygdaloid nuclei, including inhibition of alpha-2 adrenergic transmission in the amygdala, abolishes salience-induced vasoconstriction of the thermoregulatory beds (Mohammed et al. 2013,Mohammed et al. 2016).

Output from the lateral habenula also inhibits dopamine-synthesizing neurons in the ventral tegmental area (VTA) via stimulation of GABA-synthesizing neurons concentrated in the caudal “tail” of the VTA; the “dopamine brake” inhibits the mesolimbic dopamine reward system, with many psychiatric consequences (Bourdy and Barrot 2012; Fakhoury 2017; Stamatakis and Stuber 2012; Stephenson-Jones et al. 2012). Importantly, work from our laboratory shows that activation of the lateral habenula also powerfully increases BAT thermogenesis and constricts the cutaneous vascular bed (Ootsuka and Mohammed 2015). Furthermore, lesions of the lateral habenula decrease emotionally elicited BAT thermogenesis (Ootsuka et al. 2017), as do lesions in the region of the VTA itself (Shibata et al. 1996). It is notable that activation of dopamine D2 receptors in the nucleus accumbens, a major target of the VTA dopamine neurons, lowers body temperature (Grabowska and Anden 1976).

### Salience-related emotional “hyperthermia” may be physiological rather than pathological

The terms stress-induced “hyperthermia” and emotional hyperthermia suggest that the increase in temperature is a physiological abnormality. However, arousal-related increases in body and brain temperatures are likely to serve a biological purpose, a proposal readily accepted in relation to the circadian temperature rhythm, but still somewhat obscure in relation to variations in temperature during the waking period. During active attention to and exploration of the environment as part of the episodic rest-activity pattern of normal daily life, and as part of the circadian cycle, rats normally exhibit increases in body and brain temperature of magnitude only slightly less than the increases in temperature occurring in response to an intruder animal (Blessing and Ootsuka 2016; Mohammed et al. 2014; Ootsuka et al. 2009). Increased body and brain temperature occurs in response to ethologically diverse salient stimuli, including novel environments, social interaction, and addictive cues (Kiyatkin 2007, 2010). The moderate increase in brain temperature may facilitate the synaptic processing that underlies the complex cognitive and emotional processing required for successful interaction with the external environment.

The highly integrated nature of thermoregulation and salience attribution suggests that dysfunction in these two systems may be linked. Excessive salience-related increases in brain temperature could contribute to abnormal cognitive/emotional processing. The few studies that have investigated thermoregulation in unmedicated patients with schizophrenia report increased body temperature and increased resting thermoregulatory vasoconstriction, correlating with symptom severity and responsive to antipsychotic medication (Blessing et al. 2011; Heh et al. 1988; Shiloh et al. 2009). Reduction in brain temperature mediated by antipsychotic agents could contribute to their therapeutic action.

## Conclusion

Our study provides strong evidence that chlorpromazine, the classical first-generation antipsychotic drug, together with clozapine and risperidone, important second-generation antipsychotics, have vigorous, robust, dose-related inhibitory effects on cutaneous vasoconstriction and BAT thermogenesis, key components of emotional hyperthermia. This primary salience-related process has a similar biological basis in humans and in experimental animals. The doses required to inhibit experimental emotional hyperthermia in laboratory animals are reasonably similar to the doses required for optimal therapeutic effects in psychotic patients, so that similar CNS neurobiological processes may underlie both the physiological and the therapeutic actions on cognitive/emotional function. BAT thermogenesis and cutaneous vasoconstriction are relatively easy to measure in conscious freely moving experimental animals, suggesting that the emotional hyperthermia model could be used to evaluate new putative antipsychotic agents. Measurement of thermoregulatory cutaneous (hand) blood flow via infrared camera is already a robust and simple non-invasive procedure in humans. Effects on this parameter could be clinically useful. Now that it is feasible to assess emotionally induced BAT thermogenesis in humans, this fundamental metabolic process may prove to be abnormally high in subclasses of psychotic disorders.
